# Management of Older Adults with Locally Advanced Head and Neck Cancer

**DOI:** 10.3390/cancers14112809

**Published:** 2022-06-05

**Authors:** Daniel R. Dickstein, Eric J. Lehrer, Kristin Hsieh, Alexandra Hotca, Brianna M. Jones, Ann Powers, Sonam Sharma, Jerry Liu, Vishal Gupta, Loren Mell, Zain Husain, Diana Kirke, Krzysztof Misiukiewicz, Marshall Posner, Eric Genden, Richard L. Bakst

**Affiliations:** 1 Department of Radiation Oncology, Icahn School of Medicine at Mount Sinai, New York, NY 10029, USA; daniel.dickstein@mountsinai.org (D.R.D.); eric.lehrer@mountsinai.org (E.J.L.); kristin.hsieh@mountsinai.org (K.H.); alexandra.hotca-cho@mountsinai.org (A.H.); brianna.jones@mountsinai.org (B.M.J.); sonam.sharma@mountsinai.org (S.S.); jerry.liu@mountsinai.org (J.L.); vishal.gupta@mountsinai.org (V.G.); 2 Department of Otolaryngology, Icahn School of Medicine at Mount Sinai, New York, NY 10029, USA; ann.powers@mountsinai.org (A.P.); diana.kirke@mountsinai.org (D.K.); eric.genden@mountsinai.org (E.G.); 3 Department of Radiation Oncology, University of San Diego, La Jolla, CA 92110, USA; lmell@health.ucsd.edu; 4 Department of Radiation Oncology, University of Toronto, Toronto, ON M5S 1A1, Canada; zain.husain@utoronto.ca; 5 Department of Hematology and Medical Oncology, Icahn School of Medicine at Mount Sinai, New York, NY 10029, USA; krzysztof.misiukiewicz@mssm.edu (K.M.); marshall.posner@mssm.edu (M.P.)

**Keywords:** squamous cell carcinoma, head and neck cancer, older adult, geriatric, comorbidities, radiation oncology, elderly, chemotherapy

## Abstract

**Simple Summary:**

Approximately one third of patients with head and neck cancer are older adults. The number of older adults with head and neck cancer continues to rise especially as life expectancy increases. However, this population remains significantly underrepresented in clinical trials. Due to this, there is no clear consensus regarding the optimal treatment for older adults with head and neck cancer. In general, older adults are a complex cohort due to variations in functional and performance status, medical comorbidities, and medication management. Treatment for head and neck cancer involves surgery, radiation therapy, systemic therapy, or a combination. These treatments are highly demanding. Additionally, they are associated with toxicity which can be especially difficult for older adults to tolerate. This may lead to treatment interruptions and compromised outcomes. In order to understand the optimal treatment for older adults with head and neck cancer, novel predictive scores are being developed. Additionally, ongoing clinical trials are investigating less intensive treatments for older adults. This review provides an overview of current clinical data, treatment considerations, and future areas of investigation for older adults with head and neck cancer.

**Abstract:**

Thirty percent of patients with head and neck squamous cell carcinoma (HNSCC) are at least 70 years of age. This number continues to rise as life expectancy continues to increase. Still, older adults with HNSCC remain underrepresented in clinical trials, resulting in ambiguity on optimal management. Older adults are a complex patient population, often requiring increased support due to issues relating to functional and performance status, medical comorbidities, and medication management. Furthermore, in older adults with HNSCC, many of these conditions are independently associated with increased toxicity and worse outcomes. Toxicity in the older adult remains difficult to predict and to understand, and as treatment decisions are based on treatment tolerability, it is essential to understand the toxicities and how to minimize them. Novel predictive scores are being developed specifically for older adults with HNSCC to understand toxicity and to assist in personalized treatment decisions. There are clinical trials presently underway that are investigating shortened radiation courses and novel, less toxic systemic treatments in this population. In the forthcoming sections, we provide a detailed overview of the clinical data, treatment paradigms, and considerations in this population. This review provides a comprehensive overview of existing clinical data and clinical considerations in the older adult head and neck cancer population. Additionally, we provide a detailed overview of pertinent current and ongoing clinical trials, as well as future areas for investigation.

## 1. Introduction

Head and neck cancer is the sixth most common malignancy worldwide and approximately 30% of these patients are at least 70 years old [1,2,3]. The number of patients with head and neck squamous cell carcinoma (HNSCC) continues to rise, which is largely due to increasing life expectancies. In fact, between 2012 and 2016 the highest incidence of oral cavity and pharyngeal cancers were among those aged ≥70 years [4]. Additionally, in the next 20 years, the incidence of HNSCC is expected to increase by 64% in the older adult population [5]. However, this population remains underrepresented in head and neck cancer related clinical trials, constituting less than 5% of enrolled participants [6,7].

Treatment recommendations for HNSCC patients depends on many factors, including the tumor subsite, stage, and pathological characteristics. Treatment usually involves a multimodality therapy regimen consisting of a combination of surgery, radiation therapy (RT), and systemic therapy. Surgical approaches carry a higher risk of morbidity and mortality in the older adult population; additionally, older adult patients with multiple comorbidities are frequently poor surgical candidates. As a result, these patients are often offered a non-surgical approach, including definitive chemoradiation delivered over 7 weeks. These treatments can be extremely demanding for these patients, which may therefore lead to treatment interruptions, resulting in compromised outcomes. Moreover, treatment options for HNSCC can lead to permanent morbidity that can significantly compromise quality of life. Commonly affected domains include saliva production, mastication, swallow, taste, mucositis, pain, and speech. These side effects are highly relevant in all patients but may be even more important in the older adult population, where often the emphasis is on quality of life rather than overall survival (OS) [8]. Therefore, in the older adult population, factors such as medical comorbidities, performance status, and frailty— all of which can increase the probability of treatment-related adverse events—must be carefully considered when making treatment recommendations [9,10,11].

Due to the paucity of high-quality data in older adult patients with HNSCC, there is ambiguity on how to define standard therapy for this population. This uncertainty in management may lead to unnecessary overtreatment with a resultant increased risk of adverse events, or conversely, undertreatment, which can compromise patient outcomes. Clinicians should not rely on chronological age alone as this can result in over- or under-treatment. To date, there are no established guidelines to guide treatment recommendations in older adult patients with HNSCC [12]. Therefore, there is a growing interest in the head and neck oncology community to use comprehensive geriatric assessments (CGAs) to assist with clinical decision making in older adult patients. The Society for International Oncology in Geriatrics (SIOG) recommends the inclusion of core domains in the CGA, such as functional status, comorbidity, cognition, mental health status, fatigue, social status and support, nutrition, and the presence of geriatric syndromes (e.g., dementia, osteoporosis, polypharmacy) [13,14]. However, the use of such tools in routine clinical practice is resource-intensive and rarely performed in clinical practice or clinical trials for older adult patients with cancer [15].

This review provides a detailed overview of clinical data, treatment paradigms, and clinical considerations in older adult patients with HNSCC. Additionally, we provide a comprehensive overview of current and ongoing clinical trials and future areas for investigation.

## 2. Geriatric Assessments

Several geriatric assessments have been validated for identifying an increased risk of adverse outcomes in older adult cancer patients including the CGA, Geriatric 8 (G8), and other frailty indices [16,17,18,19,20]. As its name suggests, the CGA is a detailed evaluation recommended by the SIOG. However, CGA can be time- and resource-intensive to complete [17]. Subcomponents and abbreviated versions of CGA have been shown to be predictive of early death in older adult cancer patients [21]. The G8 questionnaire includes eight items: food intake, weight loss, mobility, neuropsychological problems (including dementia and depression), BMI, polypharmacy, self-reported health status, and age [16,22].

These geriatric assessments are especially important for understanding treatment for HNSCC as they can guide treatment choices. For example, a more aggressive treatment (e.g., trimodality therapy) may be selected for a fit patient and less aggressive treatment may be selected for a frail patient. The Chemotherapy Risk Assessment Scale for High-Age Patients (CRASH) is a score that can be used to identify patients at risk for chemotherapy-related adverse events [23]. Alternatively, the G8 panel has been shown to be indicative of OS and quality-adjusted survival for older adults with HNSCC patients, with a trend toward an increased length of postoperative stay and a decreased completion rate of radiotherapy in patients identified as vulnerable (G8 score ≤ 14) [24,25]. In a study evaluating how a geriatric assessment correlates with survival and quality of life outcomes, the vulnerable patients defined by the G8 had a worse OS compared to fit patients [24]. An ongoing prospective trial evaluating the use of geriatric assessment for HNSCC implemented a G8 screening tool followed by the CGA to guide intervention. Patients with locally advanced HNSCC were placed into three categories: fit, vulnerable, and frail according to the G8 and the CGA. They found that after the use of a geriatric assessment, the proposed therapy changed from what the physician was originally going to recommend in 12% of patients. Additionally, the researchers found that specific supportive needs, including nutritional, psychological, and chronic medication support, changed after the use of the geriatric assessment [26].

Still, the comprehensive assessments do not seem to be as regularly a part of practice as desired. An online survey asking radiation oncologists with expertise in HNSCC identified a gap in the management of older adult HNSCC patients. Only 10% of the radiation oncologists interviewed performed a CGA in older adult patients and approximately 7% of multidisciplinary tumor boards included a geriatrician [27]. This is problematic as another study found that for approximately 60% of cancer cases discussed with a multidisciplinary team, the diagnosis and management of the patient changed [28]. This illustrates the importance of incorporating an approachable assessment for older adult patients with HNSCC when discussing treatment options. The abovementioned studies emphasize the need to comprehensively evaluate older adult patients with a variety of metrics rather than chronologic age alone.

## 3. Medical Comorbidity

Older adult patients with HNSCC frequently have multiple medical comorbidities. Eytan and colleagues identified a large comorbidity burden in older adults with HNSCC, and the most common comorbidities included hypertension, hyperlipidemia, diabetes, and chronic obstructive pulmonary disease [29]. Given there are many possible comorbidities that can involve multiple organ systems, there is a need to quantify these comorbid conditions to determine their impact on outcomes. Multiple comorbidity measurements have been developed, including the Charlson Comorbidity Index (CCI), Kaplan Feinstein Comorbidity Index (KFI), and the Adult Comorbidity Evaluation-27 (ACE-27) [30,31,32]. Datema and colleagues have demonstrated that comorbidities impact not only OS but also the short-term mortality of HNSCC patients [33]. In older adult HNSCC cancer patients, comorbidity quantified by the CCI has been demonstrated as a predictor of survival [34]. A predictor of 10-year survival, the CCI utilizes the presence or absence of certain comorbidities, which includes cardiac, neurological, pulmonary, and gastrointestinal conditions. Three neurological comorbidities included in CCI are dementia, CVA, or TIA, which all could lead to cognitive impairment. Nonetheless, there is limited research assessing cognitive impairment in older adult HNSCC populations and its impact on outcome, with one study finding cognitive impairment in older HNSCC patients correlates significantly to postoperative delirium [35,36]. Comorbid conditions are an independent predictor of survival for OS and are important to consider in the treatment of older adult HNSCC patients [37].

## 4. Polypharmacy

Given the prevalence of comorbidities in the older adult population, polypharmacy may be an indirect measure of comorbidity burden, and thus a predictor of patient outcomes. Polypharmacy has been shown to be associated with prolonged hospitalization and post-treatment hospital readmission not secondary to cancer in older adult HNSCC patients [38]. The Centers for Disease Control and Prevention showed that approximately 90% of individuals 65 years and older overuse at least one prescription medication; in a typical month, 66% use three or more drugs, and 42% use five or more drugs [39]. This demonstrates that the majority of the older adult population in the United States is susceptible to polypharmacy. A systematic review including 47 articles analyzing older adult cancer patients showed an association of polypharmacy with chemotherapy-induced toxicities, postoperative complications, and decreasing functional status [40]. Given the concern for excessive medication utilization in the older adult population and polypharmacy, the American Geriatrics Society (AGS) regularly updates the Beers Criteria, a list of potentially inappropriate medication recommended to be regularly assessed by medical providers [41]. Prior studies have shown potentially inappropriate medication use is associated with all-cause mortality in older adult cancer patients [42]. In a retrospective study of 287 patients at least 70 years of age with oropharyngeal squamous cell carcinoma patients (OPSCC) that investigated the effects of polypharmacy, potentially inappropriate medication use, and comorbidities on outcome identified that a comorbidity-polypharmacy score—but not comorbidity score alone—was correlated with OS. This study further supports the importance of assessing medication management in older adult patients with HNSCC [43].

## 5. Treatment Toxicity

Natural physiologic changes occur with aging, including decreased muscle mass, diminished pulmonary function, and an increased risk of infection. These therefore place the older adult population at an increased risk for adverse events and prolonged recovery. In HNSCC, older adult patients are often considered to be more vulnerable to treatment-related toxicities. Toxicities associated with treatment for HNSCC (whether surgery, RT, and/or chemotherapy) are associated with significant rates of morbidity, and they are particularly concerning in the older adult population as they frequently have a high burden of preexisting medical comorbidities. Surgery may alter form and function, while the acute effects of RT or chemoradiation treatment include mucositis, dysphagia, pain, dermatitis, xerostomia, and dysgeusia can lead to treatment interruption, malnutrition, and weight loss, which may necessitate the placement of a G-tube for nutritional support. Long-term side effects of RT or chemoradiation include cavities, trismus, osteoradionecrosis, and radiation fibrosis syndrome, among others [44,45].

Multiple retrospective studies have reported either an increased or a similar radiotherapy-induced toxicity in older adult patients when compared to younger patients [7,46,47,48,49]. Meanwhile, the correlation between increased toxicity with the addition of chemotherapy in this patient population is more well established [7,46]. An analysis of three Radiation Therapy Oncology Group chemoradiation trials for HNSCC showed a significantly higher occurrence of severe late toxicity (defined as chronic grade 3+ pharyngeal/laryngeal toxicity and/or requirement for a feeding tube ≥ 2 years after registration and/or potential treatment-related death within 3 years) in the older adult group [48]. In contrast, a recent retrospective study of HNSCC patients who underwent surgery followed by adjuvant CRT compared patients ≥ 70 of age with their younger counterparts and found that chronic nephrotoxicity was the only toxicity for which a significant difference was observed between younger and older adult patients [49]. Overall, the side effects were, in general, slightly less pronounced in young patients compared to the older adult; however, the difference was marginal. One retrospective study (n = 220) of patients aged ≥ 70 with HNSCC treated curatively reported that comorbidity (measured with the CCI) did not predict toxicity [47]. In this cohort there was a higher G-tube placement than expected (24% prophylactic and 18% reactive), which is consistent with other studies, suggesting G-tubes are more frequently placed in older adult patients with HNSCC [50].

Additionally, older adult patients have been found to be at an increased risk of treatment interruption, which may be due to the treatment type as a more demanding treatment can contribute to an interruption [9,51,52]. Treatment interruptions are a concern in HNSCC patients as treatment interruptions are associated with worse tumor control [53,54,55,56,57]. In the older adult, treatment interruptions have also been associated with decreased OS [47] but not disease-related outcomes. This suggests that the etiology of treatment interruptions in the older adult population is likely multifactorial, and that it may be a surrogate for other specific issues that contribute directly to poor outcomes, such as comorbidities, socioeconomic status [57], access to insurance [55], issues with transportation [58], or issues more common to older adults such as social support [59], daily functioning [60], and psychiatric comorbidities [61]. Additionally, cancer-related weight loss, treatment-related weight loss, and age-related weight loss, termed sarcopenia, may be associated with worse toxicity and survival outcomes [47]. These are important to consider when managing an older adult with head and neck cancer.

The toxic effects following surgical management in an older adult patient may also dictate treatment for HNSCC. Despite the dramatic advances in surgical techniques, better perioperative care, and safer anesthesia over the past several decades, surgery can still lead to postoperative death and major complications [62]. Data on surgical complications in the older adult with HNSCC are limited and reports are heterogenous. While some studies reported higher risks in surgery for HNSCC in the older adult population [63,64], other studies reported frailty, a higher rate of preexisting comorbidities, an advanced tumor stage, surgery time, a poor G8 score, and smoking habits as being correlated with perioperative complications rather than chronological age [65,66,67,68,69,70,71,72]. Moreover, while free flap reconstruction is associated with longer operative times, studies have shown that older adult patients who undergo free flap reconstruction experience similar outcomes as their younger counterparts; however, they are more likely to have longer hospital stays [73,74]. Lastly, it has been shown that older adults with HNSCC undergo longer intensive care unit stays when treated at lower volume treatment centers. This illustrates that higher volume centers may be better equipped to manage toxicity associated with the treatment of HNSCC in older adults [75,76]. Toxicity in the older adult remains difficult to predict and to understand. As treatment decisions are based on treatment tolerability, it is essential to understand the potential toxicities and how to minimize them.

## 6. Novel Predictive Assessments

To better understand toxicity and management, novel predictive scores are being developed. These scores will enable personalized treatment decisions based on a risk stratification of a patient. The Cancer and Aging Research Group (CARG) geriatric assessment was developed to predict chemotherapy toxicity. This model and algorithm takes into account age, cancer type, planned chemotherapy, laboratory values, and independent activities of daily living. It was designed to understand the risk of grade 3 to 5 chemotherapy toxicity in older adults with cancer, and it was then internally validated [77].

The generalized competing risks (GCE) model is an approach to risk-stratify patients by considering the relative hazard of cancer and non-cancer related events [78,79,80,81,82,83]. The GCE is also called the ω score, which is a ratio developed for all head and neck cancer patients. The ratio is a proportion of overall event risk that is attributable to cancer and patients with a value close to 1 have a high risk of cancer progression relative to mortality from other causes, while patients with a proportion close to 0 have a higher likelihood of mortality from other causes. Patients with a ratio closer to 1 and a higher risk of death related to cancer would likely benefit from intensive cancer therapy [82]. This score has been reassessed and validated using data from patients with locally advanced head and neck cancer using 81 randomized trials from the meta-analysis of radiotherapy in squamous cell carcinomas of head and and neck (MARCH) [84,85] and the meta-analysis of chemotherapy in head and neck cancer (MACH-NC) [6,83]. Moreover, the model was validated in a cohort of older adult patients with HNSCC from the SEER-Medicare database. This study showed a more reliable way to risk stratify older adults and their risk of cancer related mortality compared to other causes [79]. Covariates included in the GCE model include age, ECOG, BMI, primary tumor site, N stage, and p16 status. Factors associated with a higher ratio and a higher likelihood to benefit from intensive therapy included younger age, better performance status, oral cavity site, and a higher T and N category p16 negative/unknown status [83]. An online tool (www.comogram.org, accessed on 30 April 2022) outlines the various predictive tools including: GCE, G8, CCI, CARG, ACE-27, and the cumulative illness rating scale-geriatric (CIRS-G). This online tool is being used a part of an ongoing clinical trial (NCT: 03258554: Radiation Therapy With Durvalumab or Cetuximab in Treating Patients with Stage III-IVB Head and Neck Cancer Who Cannot Take Cisplatin), which is investigating durvalumab or cetuximab in patients with HNSCC that are unable to take cisplatin, which includes many older adults [82].

In addition, predictive scores specific to older adults with head and neck cancers are being developed. In a retrospective study of 284 patients at least 65 years of age, researchers developed a novel predictive nomogram. The researchers then used an external cohort of 217 patients to validate the predictive model. The predictive model included KPS, CCI, and baseline serum CRP. This nomogram can hopefully help to guide treatment decisions [86]. Another group of researchers tried to identify clinical predictive indicators to prognosticate older adult patients with oral cavity squamous cell carcinoma (OCSCC). They established a predictive nomogram incorporating nodal status, hemoglobin level, body mass index, and neutrophil-lymphocyte ratio (NLR). Similar to other nomograms, the intention of this predictive model was to guide treatment for older adult patients with OCSCC. This study included 554 patients at least 60 years of age [87]. Using retrospective data from 583 patients, another group of researchers sought to develop a predictive model for older adult patients at least 65 years old with nasopharyngeal squamous cell carcinoma. They identified a predictive model that included tumor and nodal stage, EBV viral load, and albumin level to be able to risk stratify [88]. Nonetheless, these predictive models are based on retrospective data and must be validated in the prospective setting. Furthermore, it remains unclear how grade impacts clinical decision making. A comprehensive assessment of frailty and identification of relevant biomarkers that go beyond chronological age should be investigated in future studies. Nonetheless, these predictive models offer a scale, and they should function as a guide for clinical discussions with patients.

## 7. Special Consideration of Tumor Subsite

Within the various head and neck disease sites, oropharyngeal squamous cell carcinoma (OPSCC) is an important tumor subsite that is growing within the older adult population with HNSCC. The clinical and the epidemiological landscape of OPSCC continues to evolve as the HPV epidemic grows and tobacco use declines [89,90,91,92]. The rise of HPV infection has caused an increase in the incidence of OPSCC in North America and Western Europe with 70% of new OPSCC being attributable to HPV [93]. Between 2009 and 2010, the highest prevalence of HPV infection was reported in those aged 55–60 [94]; however, as the HPV+ population ages, a new wave of older patients with HPV+ OPSCC will shift the cancer burden to an older adult cohort [89,95,96]. This is supported by the fact that HPV+ OPSCC represents 70% of OPSCC cases in both the general and the older adult population [90,97]. Therefore, it is not epidemiologically accurate to associate HPV+ OPSCC with a younger cohort. However, due to its historic association with younger patients and fewer comorbidities compared to environmentally-related OPSCC, HPV+ OPSCC has been classically associated with patients with good performance status, fewer comorbidities, and longer life expectancies [98]. However, aging is associated with an onset of illness, and HPV may also be associated with age-related comorbidities including cardiovascular diseases [99,100,101]. Despite the increased prevalence of HPV+ OPSCC in the older adult, prospective data are limited. Thus, it is essential to understand how to best optimize treatment for HPV+ OPSCC patients as the population ages and the HPV epidemic evolves. HPV status has been shown to portend better clinical outcomes in HNSCC patients [43,102]. Studies have demonstrated that HPV+ status in the older adult population is associated with not only locoregional control but also OS and disease specific survival [43,90].

Thus, current de-escalation trials for HPV associated OPSCC focused on decreasing acute and chronic toxicity; although intended for younger patients with longer life expectancy, older adult patients would also benefit from enrollment in these trials. De-escalation would improve treatment compliance and tolerability in older adults. Thus, older adult patients with HPV+ OPSCC likely have superior outcomes compared to other sites, and they should be considered for de-escalation trials.

## 8. Current Trends in Management

In HNSCC, the regimens are diverse. De-escalation strategies in HPV+ OPSCC have been increasing over time; however, within the older adult population, de-escalation has not been as widely studied. Regarding de-escalation trials, Chen et al., assessed the definitive radiotherapy of 54 Gy in 27 fractions and 60 Gy in 30 fractions with 2 Gy/fractions, while Chera et al., assessed 60 Gy definitive radiotherapy after a response to induction chemotherapy [103,104]. Furthermore, Marur et al., investigated the definitive RT of 54 Gy and 69.3 Gy after determining a response from induction chemotherapy [105]. Additionally, Ma et al., determined that dose de-escalation of 30 to 36 Gy of adjuvant RT with concurrent docetaxel for patients with HPV+ OPSCC resulted in comparable locoregional control rates to historical controls receiving 60 to 66 Gy with low toxicity. This treatment was administered over 2 weeks in 1.5 Gy or 1.8 Gy twice daily [106]. These studies found that lower regimens were associated with comparable progression free survival, with a reduced toxicity profile and an improved swallowing and nutritional status.

Hypofractionation has a precedent in de-escalation therapy, where total dosage has been reduced thereby reducing the number of fractions. Because standard regimens of RT for HNSCC are associated with increased toxicity and a significant side effect profile, there is ongoing research investigating radiation dose reduction in HNSCC. It has been shown that hypofractionation and shortening total treatment time and potentially dose are satisfactory in a palliative situation to relieve patients of malignancy-associated burden. Still, the main objective of these studies was for rapid symptomatic improvement rather than local control [107,108,109,110,111,112,113].

Alternative fractionation and hypofractionation are based on the observations of Withers et al., who observed a relationship between radiation treatment time and the dose required for tumor control. This study showed that after four weeks of radiation, an increased dose of radiation was required to get the same tumor control due to tumor repopulation. This laid the groundwork for alternative fractionation schemes in head and neck cancer [114]. More recently, with the COVID-19 pandemic, the America Society of Radiation Oncology and the European Society of Radiation Oncology published a statement for recommendations of treatment of HNSCC during the COVID-19 pandemic. These societies strongly agreed that in the setting of severely reduced radiation capacity, a hypofractionated treatment regimen and schedule should be used [115]. Older adults are the perfect population for this treatment regimen given their complexity.

Hypofractionation delivered with curative intent for HNSCC has not been extensively explored, and older adult patients represent the ideal cohort to investigate its potential benefits. Radiobiological modelling showed that a 3 Gy per fraction or accelerated hyperfractionation (1.8 Gy given twice daily) is more effective for HNSCC tumor control with a reduction in late effects compared to the standard 2 Gy per fraction [116]. Nevertheless, there are a small number of studies investigating hypofractionation for older adult HNSCC patients. De Felice et al., investigated a hypofractionated radiation course given with cetuximab [117]. The median age was 77.5 years of age and the trial included 6 patients in total due to premature trial closure from toxicity. The prospective trial included a course of radiation of 60 Gy given over 4 weeks (3 Gy/fraction) with concurrent cetuximab (400 mg/m^2^ given a week before radiation followed by 250 mg/m^2^ during treatment). The primary endpoint of this study was objective response (partial or complete response at 3 months). All of the patients completed the radiation schedule while only two of the patients successfully completed concurrent cetuximab as well. A partial response was observed in three patients, while three patients had progressive disease. No patients had a complete response. However, due to toxicity, the trial was stopped early. These toxicities included grade 3+ oral mucositis, acneiform eruption, dermatitis, and nausea in 4, 3, 1, and 1 patients, respectively [117].

## 9. Ongoing Trials and Future Directions

**Figure 1:**
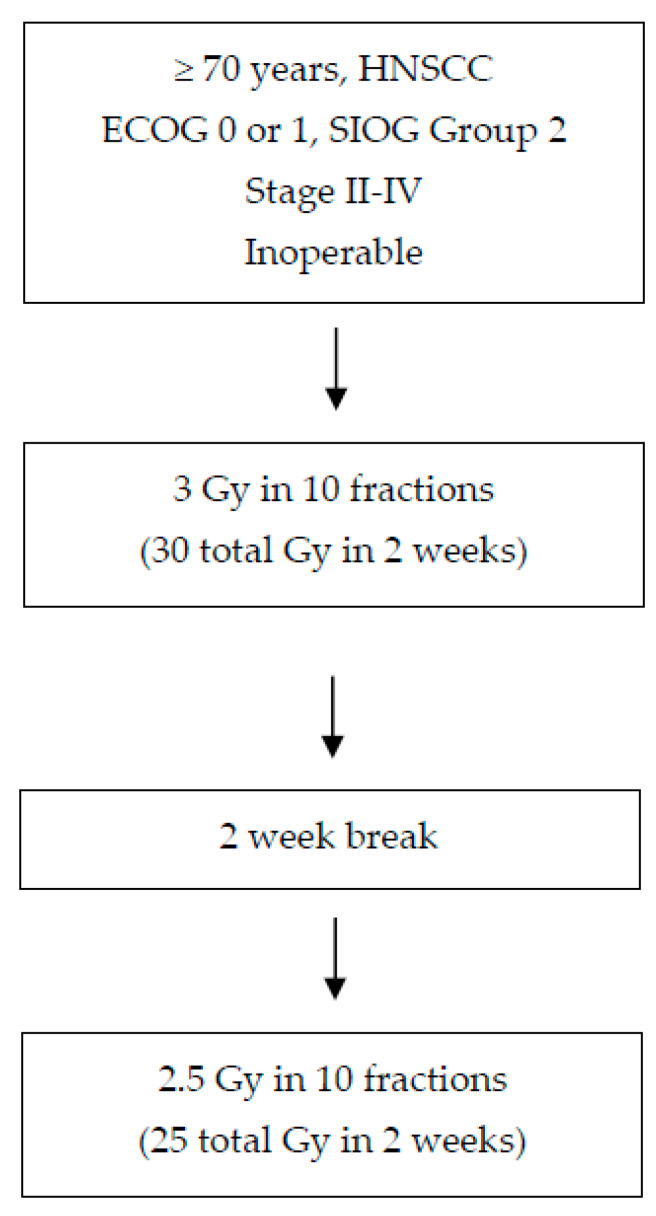
Schema of split course hypofractionation for older adult patients with head and neck squamous cell carcinoma unfit for standard treatment. For older adults with head and neck cancer that are suitable for standard treatment, consider standard treatment or enrollment onto a de-escalation trial if p16 positive.

Ortholan et al., are currently investigating a hypofractionated split-course plan in the vulnerable older adult population in the older adult head and neck (ELAN) study, one of the first prospective trials developed specifically for older adult patients [118,119]. The trial has three arms investigating novel treatment for older adult patients with head and neck cancer (unfit nonmetastatic, fit metastatic, unfit metastatic disease). Fitness was classified using a variety of geriatric assessments. For unfit patients with nonmetastatic, nonrecurrent HNSCC, patients can receive a hypofractionated split course of 30 Gy in 10 fractions over the course of 2 weeks followed by a 2 week break, then followed by 25 Gy in 10 fractions for a total of 6 weeks (Figure 1). The primary endpoint for this trial is local control at 6 months after the end of radiation. This study hopes that a split course will decrease side effects such as the incidence of radiation-induced mucositis and allow time to recover. Nonetheless, a split course poses problems as it may lose the potential efficacy of treatment due to the rapid repopulation typical for head and neck cancers after 4 weeks of treatment initiation [120]. Moreover, a split course in addition to pure hypofractionation may cause more long-term side effects [119].

**Figure 2:**
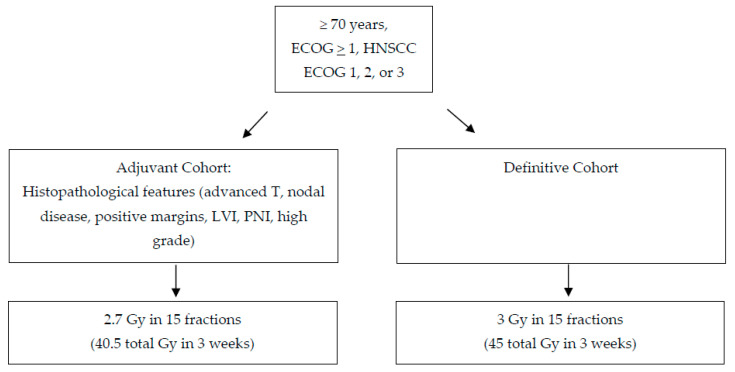
Schema of continuous hypofractionation for older adults with head and neck squamous cell carcinoma unfit for standard treatment. For older adults with head and neck cancer that are suitable for standard treatment, consider standard treatment or enrollment onto a de-escalation trial if p16 positive.

Our institution treats many older adult patients with HNSCC and our experience with unfit, frail patients is consistent with their challenge in receiving treatment [47,90,121]. For patients at least 70 years old—either surgical or non-surgical candidates—with HNSCC who cannot or do not want to receive standard RT, a new regimen such as hypofractionation is being investigated at our institution. Both standard treatment regimens—adjuvant and definitive—are shortened in order to benefit both older adult patients who can tolerate surgery and those with medical comorbidities or with unresectable tumors. The aim of the study is to evaluate the efficacy of hypofractionated RT without chemotherapy in older adult patients with HNSCC for curative intent. For this study, patients with HNSCC at least 70 years old and at least ECOG grade 1 are enrolled to receive definitive RT (45 Gy in 15 fractions over 3 weeks) or adjuvant RT (40.5 Gy in 15 fractions over 3 weeks) on a prospective trial. Inclusion criteria for the definitive arm included patients unfit for standard of care, and the adjuvant arm additionally included patients with adverse features on pathology following definitive resection. Like Ortholan et al, the primary endpoint is locoregional control at 6 months (NCT04284540: Hypofractionated Radiotherapy in Elderly Patients With Head and Neck Squamous Cell Carcinoma) (Figure 2). Investigators hypothesize that the hypofractionated treatment regimen will improve tolerance of treatment with the same efficacy.

The MACH-NC meta-analysis illustrated that there is no added benefit of chemotherapy in patients at least 70 years of age [6]. For that reason, many hospitals withhold chemotherapy in this cohort. Still, further investigation into the systemic agents for this cohort is required. Interventional clinical trials for older adults with head and neck patients are outlined in Table 1.

## 10. Conclusions

Older adult patients have historically been omitted from investigational head and neck cancer trials due to upper age limits typically set for eligibility that exclude patients at least 70 years of age. More studies are needed to fully comprehend the best treatment for older adult patients with HNSCC. As new initiatives develop, the older adult may be an ideal group to benefit from these efforts. It will be important to understand how to best optimize treatment for this understudied population. Thus, this review serves to guide such clinical trial design for older adult patients with primary HNSCC—more specifically it can help to refine treatment for older adult patients with good outcomes as well as assist older adult patients with both poor outcomes and tolerability. This review also emphasizes the importance of comprehensive geriatric assessments and the development of an appropriate surrogate marker for fitness and frailty that goes beyond chronological age.

## Figures and Tables

**Table 1:** Ongoing Clinical Trials for Older adult Patients with Locally Advanced Head and Neck Cancer.

Study Title	NCT Number
NBTXR3 With or Without Cetuximab in LA-HNSCC	NCT04892173
Hypofractionated Radiotherapy in Older adult Patients With Head & Neck Squamous Cell Carcinoma	NCT04284540
Non-Inferiority Trial of Standard RT Versus Hypofractionated Split Course in Older adult Vulnerable Patients With HNSCC	NCT01864850
Docetaxel and Radiation Therapy in Treating Patients With Stage II or Stage III Cancer of the Larynx or Hypopharynx	NCT00243113
Phase I-II Study on Treatment of Advanced Oropharynx Cancer in Older adult Population by Intensity Modulated Radiotherapy With Treatment Volume Reduction and Combination Chemotherapy	NCT01709006
Study Comparing Pembrolizumab With Methotrexate in Older adult, Frail, or Cisplatin-ineligible Patients With Head and Neck Cancers	NCT03193931
Radiotherapy, Cetuximab, and Injections of TNFerade™ Biologic for Older adult or Frail Patients With Head and Neck Cancer	NCT00496236
Phase III Trial Comparing Methotrexate and Cetuximab in First-line Treatment of Recurrent and/or Metastatic Squamous Cell Head and Neck Cancer	NCT01884623
Radiotherapy Combined With Raltitrexed Versus Radiotherapy Alone in Older Patients With HNSCC.	NCT03196843
Non Elective Vulnerable Older adult Radiotherapy (NEVER)	NCT04832555
